# Review of the Current Knowledge on the Role of Stem Cell Transplantation in Neurorehabilitation

**DOI:** 10.1155/2019/3290894

**Published:** 2019-02-25

**Authors:** Anna M. Kamelska-Sadowska, Joanna Wojtkiewicz, Ireneusz M. Kowalski

**Affiliations:** ^1^Clinic of Rehabilitation, Provincial Specialist Children's Hospital in Olsztyn, 18A Żołnierska Street, 10-561 Olsztyn, Poland; ^2^Department of Pathophysiology, School of Medicine, Collegium Medicum, University of Warmia and Mazury, 30 Warszawska Street, 10-082 Olsztyn, Poland; ^3^Department of Rehabilitation, School of Medicine, Collegium Medicum, University of Warmia and Mazury, 18A Żołnierska Street, 10-561 Olsztyn, Poland

## Abstract

The management involving stem cell (SC) therapy along with physiotherapy offers tremendous chance for patients after spinal cord injury (SCI), traumatic brain injury (TBI), stroke, etc. However, there are still only a limited number of reports assessing the impact of stem cells (SCs) on the rehabilitation process and/or the results of the simultaneous use of SC and rehabilitation. Additionally, since there is still not enough convincing evidence about the effect of SCT on humans, e.g., in stroke, there have been no studies conducted concerning rehabilitation program formation and expected outcomes. It has been shown that bone marrow-derived mesenchymal stem cell (BMSCs) transplantation in rats combined with hyperbaric oxygen therapy (HBO) can promote the functional recovery of hind limbs after SCI. An anti-inflammatory effect has been shown. One case study showed that, after the simultaneous use of SCT and rehabilitation, an SCI patient progressed from ASIA Grade A to ASIA Grade C. Such promising data in the case of complete tetraplegia could be a breakthrough in the treatment of neurologic disorders in humans. Although SCT appears as a promising method for the treatment of neurological conditions, e.g., complete tetraplegia, much work should be done towards the development of rehabilitation protocols.

## 1. Introduction

Human pluripotent embryonic stem cells (ESC) were first isolated by Dr. James Thomson (PhD, VMD), who is perceived to be the founder of the ESC concept [1]. This was a great scientific breakthrough, which transformed medicine [2]. Previously, Joseph Altman (PhD) of the Massachusetts Institute of Technology (MIT) discovered neuronal generation (neurogenesis) in rats in 1962. This was the starting point for the discovery of a new multipotent line of stem cells (SCs) which were found in the brain (neural stem cells, NSCs) [2].

Stem cells are immature cells which have the unique property of self-renewal and differentiation into multiple cell types [3]. SCs are already a part of the human repair system. After reaching their specific site of destination, they could replace the damaged cells and the restoration of the brain develops.

Until recently, it has been believed that damage to brain tissue is permanent but the regrowth of brain cells and improvement of neurological function have now been documented. In fact, a growing number of reports indicate that adult stem cells [e.g., neural stem cells (NSCs) in the brain] have the ability to stimulate the generation of three major cell types, new neurons, and two categories of nonneuronal cells: oligodendrocytes (e.g., by direct lineage conversion) and astrocytes. The increased proliferation of neural stem cells (NSCs) was proved to come from endogenous neural progenitor cells. Moreover, these data suggest that implants of exogenous NSCs may promote regeneration in aging organisms through stimulation of endogenous neurogenesis [4, 5].

Recently, the molecular mechanisms involved in the process of differentiation of the NSCs have been described [6]. The creation of a new functional neuron includes the self-renewal of neural stem cells and neural precursor cells, the generation of neuroblasts that differentiate into young neurons that migrate, mature, and integrate into the preexisting neuronal circuit, processes regulated by the dynamic interaction between the genome, epigenetic mechanisms, and extrinsic signals. Among the transcription factors, Tlx orphan nuclear receptor is essential for the maintenance and self-renewal of NSCs in adult brains. Additionally, it has been shown that the activation of estrogen receptors by 17 beta estradiol (E2) regulates the proliferation of embryonic NSCs mediated by overexpression of the cyclin-dependent kinase inhibitor, p21Cip1 [7], while promoting the proliferation and differentiation to glial cells of NSC embryonic rat in the absence of mitogens epidermal growth factor (EGF), fibroblast growth factor-2 (FGF-2), or differentiation factors [8].

Moreover, treatment involving stem cell (SCs) therapy combined with physiotherapy (as a supportive therapy) offers a tremendous opportunity for patients with neurological disorders, e.g., after spinal cord injury (SCI) [9] (Jin et al., 2016), traumatic brain injury (TBI) [10] stroke [11], etc. The rehabilitation itself could prevent the process of muscle atrophy and joint stiffness, but it cannot repair the damaged nerve function. On the other hand, it was also shown that in adult rats physical activity increases the proliferation of endogenous stem cells in injured spinal cord tissues [12].

The most commonly used cells in therapy are embryonic stem cells from the blastocyst, neural stem cells from the embryonic or adult brain, or stem cells collected from other tissues, e.g., from bone marrow.

Since the route of stem cell delivery into the central nervous system (CNS) still remains a challenge, the intranasal (i.n.) delivery of stem cells could be beneficial. In addition, migration of SCs from the nasal mucosa into the general blood circulation cannot be excluded and the migration within the brain might be confirmed [13]. Intranasal delivery of stem cells might therefore be a safe and noninvasive method of targeting the CNS and would thus be a promising therapeutic option for CNS diseases [14].

In addition, rejuvenation of many body tissues occurs during SCT. Moreover, rejuvenated niches could rejuvenate the stem cells already residing within them, thus making all of the organs healthier [15]. This change comes from the modulation of signaling pathways. The modulation of signaling pathways such as Notch/Delta, Wnt, transforming growth factor-*β*, JAK/STAT, mammalian target of rapamycin, and p38 mitogen-activated protein kinase has demonstrated potential to rejuvenate stem cell function leading to organismic rejuvenation. Several synthetic agents and natural sources, such as phytochemicals and flavonoids, have been proposed to rejuvenate old stem cells by targeting these pathways.

The success of regenerative processes is limited by the aging of the niche and the systemic environment, but also SCs themselves. New strategies may include identifying and using immune cell-derived factors that stimulate a specific aspect of the regenerative process or targeting the immune cells themselves with instructive signals to modulate regeneration. Specific environmental niche components, including growth factors, ECM, and immune cells, and intrinsic stem cell properties will be critical for development of new strategies to improve stem cell function and optimize tissue repair processes [16].

In most SCT cases, regenerative rehabilitation has been implemented. It integrates regenerative technologies with rehabilitative clinical practices to restore function and quality of life in individuals with disabilities due to otherwise irreparable tissue or organ damage caused by disease or trauma [17]. Rehabilitation programs have been directed to optimize posttransplantation recovery in support of the view that exercise and mechanical stimulation play a role in the success of musculoskeletal regeneration [18]. Numerous rehabilitation clinics have investigated the effect of stem cell transplantation on the regeneration of the intervertebral disk [19] and on the restoration of cells of the nervous system [20]. Boninger, Wechsler, and Stein [21] proved that the use of bioengineering, robotics, and stem cells may provide synergy when coupled together with regenerative rehabilitation strategies.

Neurological disorders and neurodegenerative conditions may result in paralysis, muscle weakness, poor coordination, loss of sensation, seizures, confusion, pain, and altered levels of consciousness. Motor control exercises and potentially manual therapy could induce positive changes in the central nervous system (CNS) [22]. Because of this, research has been conducted using different rehabilitation methods, such as the proprioceptive neuromuscular facilitation concept (PNF) [23], the Bobath method [24], neurobiofeedback [25], and video/computer-based interactive exercises [26].

Human stem cells provide new opportunities for the rehabilitation of patients with neurological deficits and other neurodegenerative conditions. However, experience concerning the use of stem cells and their impact on the rehabilitation process or the simultaneous use of SCs and rehabilitation approaches is still very limited. Thus, the aim of the study was to investigate the effect of physiotherapy in poststem cell transplantation patients.

## 2. The Use of Stem Cells in the Rehabilitation of Main Neurological Conditions such as Stroke, ALS, and SCI

The use of stem cells in the rehabilitation of patients with neurological disorders falls within the scope of interest of many researchers. Preliminary studies of clinical trials show that mesenchymal stem cell transplantation can remarkably improve the neurological function of SCI in animals without any severe side effect [27]. Other cell types such as primary fetal tissues and more recently neural stem cells (NSCs) have been applied in cell transplantation-based therapeutic approaches to stroke, SCI, ALS (amyotrophic lateral sclerosis) [28], and other neurodegenerative disorders in humans [29]. Additionally, mesenchymal autologous stem cells (MSCs) have been used for patients with spinal cord injuries and stroke [11, 30].

The use of MSCs in stroke survivors, ALS, and SCI patients was implemented after discovering the capacity of these cells to secrete a large variety of bioactive molecules such as growth factors (e.g., IGF-1; VEGF), cytokines (which induce suppression of the immune response, e.g., IL-6, IL-10, and TGF-*β*), and chemokines (CXCL12 and probably CCL27 and CCL21) leading to the reduction of local inflammation. Many types of CNS disorders, including brain trauma, ischemia, and SCI, are accompanied by neuroinflammation [31, 32]. It is a pathological process in which the activation of microglia and astrocytes by inflammatory mediators occurs. Moreover, MSCs are also responsible for the increase in neurogenesis from the germinative niches of the central nervous system and an increase in angiogenesis and affect the survival of astrocytes associated with glial fibrillary acidic protein (GFAP) downregulation [30]. Furthermore, MSCs enhance the survival and myelinating abilities of allogeneic oligodendrocytes in the brains of adult immunocompetent shiverer mice [11, 33].

Bone marrow-derived mesenchymal stem cells (BMSCs) have been used to treat patients with injured spinal cords. The advantages of cellular transplantation strategies for patients suffering from SCI have been evaluated, and an absence of a clinical progression of damage after a mean of 49 months was found in 75% of them [34].

However, the success of other cell types, e.g., neural stem/progenitor cell (NSPC) transplantation, depends on injury model, intervention phase, transplanted cell count, immunosuppressive use, and perhaps also a source of stem cells. The highest improvement as a result of NSPC transplantation was observed in transection and contusion models and in the acute phase of spinal injury [35]. Moreover, transplantations of olfactory ensheathing cells and of Schwann cells or a combination of them for the treatment of chronic complete spinal cord injuries have been well-tolerated and have beneficial effects in patients [36].

The above-mentioned neurological conditions and stem cell therapies provide promising results. It has been shown that stem cell transplantation could also be used as a potential new therapy for patients with ALS [27, 37]. However, during the 1-year follow-up after stem cell transplantation (autologous bone marrow-derived hematopoietic stem cell transplantation), an improvement was shown in nine of thirteen patients with ALS, and one patient was stable with neither decline nor improvement in his status. Obviously, not all ALS patients benefit from this therapy, as the three patients died 1.5, 2, and 9 months after transplantation as a result of lung infection and myocardial infarction (MI) [27]. This could be associated with a number of important considerations that must still be addressed to support stem cell therapies, e.g., elucidating the proper approach to deliver or target cellular therapies to regions where it will have maximal benefit in ALS patients. Moreover, confirmation of graft survival is imperative to achieve sustained efficacy and specific requirements for immunosuppression [38].

SCT has also found application in conditions that manifest themselves with neurological disorders, e.g., ischemic stroke. In such cases human umbilical cord-derived mesenchymal stem cells (hUC-MSCs) may act as a potential therapy. hUC-MSCs were used as a protective agent in middle cerebral artery occlusion (MCAO) mice by TGF-*β* modulating peripheral immunoinflammation. Thus, the hUC-MSCs may be a potential therapy for ischemic stroke [39].

In cerebral ischemia animal models, an immortalized human NSC clone HB1.F3 provided neuroprotection and did not affect necrotic cell death, possibly through the regulation of early inflammatory events [40].

Recently, the transplantation of Noggin-modified bone marrow stromal cells (BMSCs) and/or brain-derived neurotrophic factor (BDNF) has also been reported as a potential therapeutic method for ischemic stroke in clinics [41].

Not only the cell type but other factors have influence on the success of SCT. Higher doses of SCs (>3 × 10^6^ cell/kg) were shown to be optimum for transplantation, but immunosuppressive agent administration negatively affected the motor function recovery.

A biomaterial scaffold synthesized from either a natural or synthetic polymer can help prevent the formation of scar tissue and concentrate neurotrophic growth factors while promoting axonal regeneration between the two ends of the injured neural tissue. To enhance axonal growth, biological molecules, such as full-length proteins or shorter peptide chains, have been conjugated on the surface of the scaffold to mimic a natural extracellular matrix [53]. The use of a biomaterial scaffold in NSPC transplantation could also effectively raise functional recovery, by improving cellular activities [36].

In conclusion, these findings suggest that optimization of the cell dose, the timing, and route of administration as well as the role of biomaterials are critical to the success of SCT in neurorehabilitation.

## 3. Rehabilitation after/along with SC Transplantation

The physical activity of people with spinal cord injury (SCI), stroke and other neurological disorders were lower compared to the general population and also lower than people with other chronic diseases [54–56]. Patients suffering from stroke mostly had a sedentary lifestyle and their inactivity not only decreased physical performance, but also contributed to the recurrence of cardiovascular disease and even subsequent incidence of stroke [57]. The physical activity of ALS patients with severe symptoms was less regular. Moreover, their nutritional status as assessed by the body mass index (BMI) and geriatric nutritional risk index (GNRI) was lower and the intake of nutrients decreased with the progression of the disease [58].

It is even more important because it has been shown that physical activity stimulates neural plasticity in animal models. The increase in the number of astrocytes and neural stem cells in the lower granular cell layer of the dentate gyrus in mature rat hippocampus has been confirmed. It has been shown that exercise stimulates the proliferation of endogenous neural stem cells and generates neurotrophic factors, such as* brain-derived neurotrophic factor* (BDNF), which in turn regulate neural plasticity and improve motor function [59, 60].

Increasing the amount of physical activity for patients presenting with SCI was shown to reduce the risk of cardiovascular disease, prevent or reduce secondary health problems such as pressure areas, and improve physical fitness and quality of life [61]. Considering the latter, more research is needed to verify the effect of various types of rehabilitation on the survivors of neurological deficits. However, at this early stage of stem cell transplantation, data concerning the use of stem cells and rehabilitation programs simultaneously are even more limited.

Rehabilitation procedures should be based on good diagnoses and the evaluation of patient conditions, which determines the use of a method optimal in each individual situation. For the vast number of diseases and injuries that affect the nervous system, currently the most effectively used rehabilitation methods are the PNF concept, the Bobath method, and biofeedback. For spinal injuries, the patient's rehabilitative status could also be improved by restorative neurology and regenerative medicine (stem cell transplantation) [62]. These rely on improving residual functions through selective structural or functional modifications of insufficient neurocontrol according to the underlying mechanisms and clinically yet unrecognized residual functions [63]. The reactivation, modification, and stimulation of residual nerve fibers could also be a promising method for complete spinal injuries, which have poor chances of improvement. Regenerative therapy could serve as the treatment of choice for patients with SCI.

Evidence based medicine (EBM) recommends that the treatment choices need to be based on research facts. It is necessary to determine the possibilities and the effects of new methods. One should follow the research and work with patients according to the latest studies.  Table 1 shows the actual studies made in the field of neurorehabilitation after stem cell transplantation. The main results have also been indicated.

However, there are still limited data comparing the results of physiotherapy and regenerative medicine itself. In the case of stroke, there is still not enough convincing evidence concerning the effects of SCT in humans [64, 65], so studies regarding rehabilitation programs and expected outcomes in this case are not yet available.

Rehabilitation after stem cell transplantation aims at maintaining the current level of mobility before and after the procedure. Specific rehabilitation programs, e.g., RESTORE, have been introduced for stem cell transplant patients (mainly with cancer) who have received an allogeneic (not self) transplant. RESTORE was launched in September 2011 in the Vanderbilt-Ingram Cancer Centre [66]. It is comprised of walking on a track, resistance training, and cardiovascular training, as well as yoga, tai chi, ai chi (gentle water exercise), and mindfulness classes.

Another study (the EXIST) was aimed at introducing a specific 18-week exercise program (similar to the program developed by [67]) for patients with multiple myeloma or (non)Hodgkin's lymphoma treated with high dose chemotherapy and autologous stem cell transplantation [68]. This program consists of high-intensity resistance and interval training described in Table 2.

Rehabilitation programs for patients with cancer are becoming widely available in Germany, where similar rehabilitation has also been used in cases of cancer patients receiving SCT [69]. Cancer treatment could induce some neurological disorders in which peripheral weakness disrupts function. The treatment for patients with side effects following hematopoietic stem cell therapy (HSCT), e.g., neuropsychological deficits, was introduced in Germany [70]. It was found that in some cases splinting and orthoses were helpful [71].

The latest research by Geng et al. [72] showed that BMSC transplantation can promote the functional recovery of rat hind limbs after SCI. Mesenchymal stem cell transplantation was combined with hyperbaric oxygen therapy (HBO), and the synergistic effect of those methods on rehabilitation in animal models was confirmed. The behavioural evaluation and histopathology showed greater recovery in the combined group in comparison to HBO or MSC treatment alone. Moreover, the anti-inflammatory effect was shown as a decrease in TNF-*α*, IL-1*β*, Il-6, and INF-*α* in tissue from the focal area determined by ELISA. The combined treatment also had a better effect on recovering the electrophysiological abnormalities from hind limbs to head [72].

In humans, there is one leading report concerning a complete rehabilitation program (initial, postinjury/adaptation, and posttransplantation) in a chronic complete C4 tetraplegic [62]. The patient received postinjury treatment, which was provided over a period of three months, consisting of 60-minute sessions with a student biokineticist twice weekly. These sessions included training on an active-passive upper limb trainer and passive-assisted weight training using a pulley system. The detailed rehabilitation schedule for the first 56 weeks following autologous human stem cell transplantation (AHESC) has been described [Table II in [62]].

Rehabilitation after stem cell transplantation (during 12 months) consisted of three phases in accordance with the recovery of different muscles and function. Five days after transplantation, the patient had clinically confirmed C4/5 complete tetraplegia and an ASIA (American Spinal Injury Association) score of 29/114 (Grade A). During the first 16 weeks, the aim of physiotherapy was to maintain the proper respiratory management, joint range of motion, and flexibility. Neuromuscular rehabilitation (the same as for stroke patients) using the potential of mirror neurons [73]) and stimulation techniques such as sweeping, tapping, ice and electrical stimulation as well as computer-based programs and afferent feedback were used. After this, an improvement in diaphragm and intercostal activity was observed (recovery of the function of trunk muscles). During the second phase (at 17-40 weeks) bed mobility, upper limb use and balance during transfers, and work on a mat and a therapy ball were introduced. In the third phase, pelvic movement, a 4-point-kneeling potion with support, a tilt table, and others were used. After the treatment, the patient progressed from ASIA Grade A to ASIA Grade C. Sensation had improved from 25% (ASIA 29/114) to 60% (ASIA 69/114). The patient showed improved motor activity, sensory and vascular function, self-care, and wheelchair use as well as safety and leisure participation.

## 4. Conclusions

Stem cell transplantation appears to be a promising method for the treatment of patients with neurodegenerative conditions, spinal cord injury, and stroke. Cellular therapies and neurorehabilitation yield better outcome in comparison with single strategies. However, still not much evidence concerning the effects of rehabilitation after SCT has been shown in the case of stroke, ALS, or SCI patients. Recently reported confirmation of successful rehabilitation following SCT in patients with complete tetraplegia could be a breakthrough and provide guidance for the development of treatment and rehabilitation approaches for patients with a larger spectrum of neurological disorders. Therefore, investigating combinations of stem cell transplantation followed by various types of rehabilitation methods and the reporting of well-documented individual cases should be encouraged.

## Figures and Tables

**Table 1:** The effects of neurorehabilitation in stem cell transplantation (SCT).

Study	Neurological condition	Methods of SCT	Organism (N)	Results
[42]	chronic ischemic stroke	bone marrow-derived mononuclear stem cells (BM-MNC)	human (N=20)	Neurorehabilitation regime and SCT could increase the release of growth factors: vascular endothelial growth factor (VEGF) and brain-derived neurotrophic factor (BDNF) in the microenvironment.

[43]	left thalamic haemorrhagic stroke	autologous bone marrow stem cells	human (case study)	Exercise enhanced the effect of stem cells by helping the mobilization of local stem cells and encouraging angiogenesis. Hence, the concept of neuroregenerative rehabilitation therapy (NRRT) endeavours to combine the impact of neuroregeneration and rehabilitation for a better therapy outcome.

[44]	progressive muscular dystrophy	bone marrow and umbilical cord blood mesenchymal stem cells	human (N=82)	The combination of various therapies: cellular therapies (stem cells) and exercise (neurorehabilitation and neurofacilitation) together yield better outcome than single strategies employed independently.

[45]	muscular dystrophy, spinal cord injury (SCI), cerebral palsy (CP)	autologous bone marrow stem cells	human (N=71)	Stem cells transplantation (SCT) with individually planned neurorehabilitation gave subjective and functional improvement (in 97% of muscular dystrophy cases, in 85% of CP cases), and improvement with respect to muscle strength, urine control, spasticity (all spinal cord injury cases).

[46]	chronic spinal cord injury	neural stem cells	mice (N=80)	The neural stem cell transplantation combined with treadmill training significantly improved spinal cord pathway conduction and increased central pattern generator activity, resulting in significantly improved motor function.

[47]	spinal cord injury (SCI)	human embryonic stem cells (hESC)	human (paraplegic N=136; tetraplegic N=90)	The physiotherapy aided in training of cells and atrophy of limbs, whereas hESC therapy resulted in an overall improvement of the patients with SCI. The hESC therapy along with physiotherapy which addresses the regeneration that is progressing in the patient could herald a new approach in the treatment of SCI.

[48]	spinal cord injury	neural precursors and mesenchymal stem cells	mice (N=44)	The cotransplantation of neural precursors and mesenchymal stem cells can assure a remarkable anatomical and functional recovery following SCI, and such recovery is only partially boosted by enriched environment/exercise.

[49]	spinal cord injury	natural proliferation and phenotypical changes of ependymal cells	rats (N=51)	Physical activity and increased mobility caused the recruitment of progenitors (an increased number of nestin immunoreactive ependymal cells).

[50]	spinal cord injury	autologous bone marrow stem cells (CD45^+^/CD34^−^)	rats (N=55)	The combination of bone marrow stem cell therapy (CD45^+^/CD34^−^) and exercise training (swimming) resulted in significant functional improvement in acute spinal cord injury.

[51]	amyotrophic lateral sclerosis (ALS)	foetal stem cells (FSCs)	human (N=30)	Combined treatment of ALS including the individual program with a complex of kinesiotherapy, respiratory gymnastics and administration of FSCs suspensions proved to objectively inhibit a progression of ALS over the period from 6 to 18 months from the beginning of treatment and contributes to longer life expectancy among the patients.

[52]	amyotrophic lateral sclerosis (ALS)	autologous bone marrow mononuclear cell (BM-MNC)	Human (case study)	Cellular transplantation along with intensive rehabilitation resulted in slowing of the disease progression, and improvements in neurological symptoms.

N: number of organisms.

**Table 2:** The structure of the EXIST exercise program (Persoon et al., 2010).

Week	Type of training	Number of training sessions	Aims of the training
1-12	Resistance training and interval training (2 x 8 minutes)	2 x per week	(1) become familiar with exercise program
		60 minutes	(2) overcoming the fear of physical activity
			(3) improve coordination and muscle hypertrophy and improving muscle force
			(4) increasing aerobic capacity
			(5) increasing the pleasure in being physically active

13-18	Resistance training and interval training (2 x 8 minutes)	1 x per week	(1) maintain muscle force
		60 minutes	(2) improve muscle endurance and aerobic capacity

1,4,10, 12, 18, 22	Counseling	6 sessions of 5 to 15 minutes	(1) improve compliance to the exercise intervention
			(2) encourage patients to pursue and active lifestyle

## Abbreviations

ALS: Amyotrophic lateral sclerosis
ASCs: Autologous adipose-derived stem cells
BDNF: Brain-derived neurotrophic factor
BMI: Body mass index
BMSCs: Bone marrow-derived mesenchymal stem cells
BM-MNC: Bone marrow-derived mononuclear stem cells
CNS: Central nervous system
CP: Cerebral palsy
EBM: Evidence based medicine
ESC: Embryonic stem cells
FSCs: Fetal stem cells
GNRI: Geriatric nutritional risk index
HBO: Hyperbaric oxygen therapy
hESC: Human embryonic stem cells
i.n.: Intranasal
ICF: International Classification of Functioning
MIT: Massachusetts Institute of Technology
MSCs: Mesenchymal autologous stem cells
NRRT: Neuroregenerative rehabilitation therapy
NSCs: Neural stem cells
NSPC: Neural stem/progenitor cell
PNF: Proprioceptive neuromuscular facilitation concept
SCI: Spinal cord injury
SCs: Stem cells
SCT: Stem cell transplantation
SUC: Subtyped as of undetermined cause
TBI: Traumatic brain injury
TSCI: Traumatic spinal cord injury
VEGF: Vascular endothelial growth factor
VHMN: Ventral horn motor neuron
VMD: Veterinariae Medicinae Doctoris
WHO: World Health Organization.
